# pH-Sensitive Degradable Oxalic Acid Crosslinked Hyperbranched Polyglycerol Hydrogel for Controlled Drug Release

**DOI:** 10.3390/polym15071795

**Published:** 2023-04-05

**Authors:** Bianca Andrade de Campos, Natalia Cristina Borges da Silva, Lucas Szmgel Moda, Pedro Vidinha, Lígia Passos Maia-Obi

**Affiliations:** 1 Center of Engineering, Modelling and Applied Social Sciences, Federal University of ABC, Avenida dos Estados, 5001, Santo André 09210-580, SP, Brazil; bianca.andrade@aluno.ufabc.edu.br (B.A.d.C.); nataliacrborges@gmail.com (N.C.B.d.S.); lucas.szmgel@gmail.com (L.S.M.); 2 Department of Fundamental Chemistry, Institute of Chemistry, University of São Paulo, Avenida Professor Lineu Prestes, 748, São Paulo 05508-000, SP, Brazil; pvidinha@iq.usp.br

**Keywords:** pH-sensitive hydrogel, hyperbranched polyglycerol, hydrogel formation, controlled release

## Abstract

pH-sensitive degradable hydrogels are smart materials that can cleave covalent bonds upon pH variation, leading to their degradation. Their development led to many applications for drug delivery, where drugs can be released in a pH-dependent manner. Crosslinking hyperbranched polyglycerol (HPG), a biocompatible building block bearing high end-group functionality, using oxalic acid (OA), a diacid that can be synthesized from CO_2_ and form highly activated ester bonds, can generate this type of smart hydrogel. Aiming to understand the process of developing this novel material and its drug release for oral administration, its formation was studied by varying reactant stoichiometry, concentration and cure procedure and temperature; it was characterized regarding gel percent (%gel), swelling degree (%S), FTIR and thermal behavior; impregnated using ibuprofen, as a model drug, and a release study was carried out at pH 2 and 7. Hydrogel formation was evidenced by its insolubility, FTIR spectra and an increase in T_d_ and T_g_; a pre-cure step was shown to be crucial for its formation and an increase in the concentration of the reactants led to higher %gel and lower %S. The impregnation resulted in a matrix-encapsulated system; and the ibuprofen release was negligible at pH 2 but completed at pH 7 due to the hydrolysis of the matrix. A pH-sensitive degradable HPG-OA hydrogel was obtained and it can largely be beneficial in controlled drug release applications.

## 1. Introduction

pH-sensitive hydrogels (PSHs) are a class of hydrogels that can change their physicochemical or chemical behavior in response to changes in pH [1,2,3,4,5]. For instance, a variation in the pH of the surrounding environment can cause a conformational change that makes these materials swell or shrink [6,7], or promote cleavage of covalent bonds, resulting in a pH-sensitive degradation of the hydrogel [5,8]. The discovery of this type of material has led to the development of many applications, especially in drug delivery, where the drugs can be released in a pH-dependent manner [4]. Many types of pH-sensitive hydrogels can readily be found in the literature, including copolymer hydrogels, polyelectrolyte hydrogels or even interpenetrating network hydrogels. The most common materials used to fabricate this type of hydrogel include chitosan, alginate, hyaluronic acid, poly(acrylic acid) and poly(vinyl alcohol), among others [9,10,11,12,13,14,15]. Nevertheless, the types of materials used in producing PSH, as well as the crosslinker choice, have a dramatic impact on their response to the environmental pH and consequently on the way that the delivery process occurs. Therefore, the development of novel materials that can be used to modulate the pH-sensitive response in hydrogels is still a hot topic in the drug delivery field.

Hyperbranched polyglycerols (HPGs) have been explored as a building block in developing materials for biomedical applications [16,17,18,19,20,21,22,23]. The great interest by scientists in this polymer is due to its biocompatibility, non-immunogenic nature, good water solubility, versatility and high end-group functionality provided by its hyperbranched structure [19,21,22,23,24,25]. Moreover, HPG can be used as a substitute for PEG (polyethylene glycol) [22], due to their similarity, and they have better resistance to protein adsorption than the latter [26]. The presence of a large number of hydroxyl groups on the surface of the HPG molecules allows it to form hydrogels through the reaction with a great variety of crosslinking agents and functionalization reactants in different degrees, producing materials with a wide range of properties [17,27]. For instance, degradable hydrogels based on HPG, which are pH [28] and redox [29] responsive, could be formed using boronic acid and disulfide derivatives, respectively. Indeed, the use of crosslinking strategies that generate cleavable bonds is a key strategy for creating degradable hydrogels, which are suitable for the controlled release of drugs involving degradation and erosion of the impregnated matrix.

Ester bonds are hydrolysable bonds that can be used to obtain hydrogels whose degradation is pH-sensitive, as the speed of ester hydrolysis depends on pH [30]. Dicarboxylic acids are often used to crosslink different polymers such as alginate, chitosan, proteins and polyvinyl alcohol (PVA) [31,32,33,34,35,36]. Among dicarboxylic acids, oxalic acid (OA) is one of the most used to perform the crosslinking of such polymers since it is a naturally based agent present in dark-green leafy food, presenting a high biocompatibility which is an essential requisite for food and wealth applications [37]. Additionally, OA forms ester linkages that are highly activated for hydrolysis at neutral and basic pH [12,13], easily responding to the pH variation. Moreover, OA, when compared with other dicarboxylic acids, is capable of improving the material’s mechanical strength. For instance, Moghadas et al. have shown that the chitosan membranes crosslinked with oxalic acid had higher Young’s modulus (~1042 N·mm^−2^) and ultimate tensile strength (~75 N·mm^−2^) when compared with other dicarboxylic acids [32]. Finally, one of the merits of using OA is that it can be directly obtained from CO_2_, which allows the storage of an amount of CO_2_ in this type of material, thereby reducing their carbon footprint [38]. These are the reasons why we chose OA as the crosslinking agent to produce the HPG hydrogel. Moreover, when crosslinked by polyacids, HPG does not need the previous functionalization commonly seen in other synthetic strategies [27].

In light of this, the formation of a novel OA crosslinked HPG hydrogel (HPG-OA) and the effect of two pHs on its drug release were studied using ibuprofen as a model drug and aiming oral drug administration. A new material whose drug release can be triggered by pH change due to its degradation was obtained through an easy route and represents a great potential for controlled drug release applications.

## 2. Materials and Methods

### 2.1. Materials

HPG (Mn = 949 g mol^−1^, Đ = 1.1, 14.6 OH per mol) was previously synthesized as described by Sunder et al. [20]. Oxalic acid dihydrate (OA, 99.5%, Synth, Diadema, Brazil), ibuprofen (IBU, DEG—lmportação de Produtos Químicos Ltda, São Paulo, Brazil) and phosphate buffer pH 7 (Tecnopon, Piracicaba, Brazil) were used as received. Hydrochloric acid (HCl, 37%, Sigma Aldrich, St. Louis, MO, USA) was used as received to prepare the HCl solutions. The water was distilled prior to use.

### 2.2. Study of Hydrogel Formation

The hydrogel formation was studied by varying the stoichiometry of the reactants, the concentration of the reaction media, the time of the pre-cure and the cure steps, and the cure temperature. A total of 0.5 g of HPG and 0.05 or 0.1 g of OA (10 or 20% *w*/*w* with respect to the weight of the polymer, corresponding to 1:1 or 2:1 mol of OA:mol of HPG) were solubilized in 0.1 mL of a solution of HCl 0.05 mol L^−1^. Distilled water was added to result in reaction media with volumes from 1 to 4 mL, corresponding, respectively, to media concentration in terms of HPG ([HPG]) of 0.13 to 0.5 g mL^−1^, which was kept under stirring at 70 °C for 1.25 to 18 h (pre-cure step), poured into silicone molds of 1.5 × 3.5 cm and cured in an oven for 2.5, 13.5 or 24 h at 60 or 80 °C. Table 1 summarizes for each sample the parameters that were varied. A small aliquot of each obtained sample had the solubility in water tested in 2 mL microtubes. The resulting hydrogels were washed three times by immersion in water for 1 h to remove unreacted molecules and subsequently dried in an oven at 60 °C for 24 h. Samples 6 and 9 were selected for further analyses and named HPG-OA1 and HPG-OA2, respectively.

#### Measurement of Gel Percent

The gel percent (%gel) was determined for the samples HPG-OA1 and HPG-OA2. The weights of the dry samples before (W_i_) and after (W_f_) the washing step were determined and the %gel was calculated using the following equation:

(1) $$\%gel=\frac{W_{f}}{W_{i}}\times 100$$

### 2.3. Optical Microscopy

Morphological analysis of the hydrogel HPG-OA1 was performed in Zeiss AXIO Scope.A1 optical microscope in transmission mode to qualitatively assess the porosity and crystallinity of the hydrogel.

### 2.4. Fourier Transformed Infrared Spectroscopy

The hydrogels were chemically characterized via Fourier transformed infrared spectroscopy (FTIR) using a Spectrum Two FTIR spectrometer (PerkinElmer), operating in ATR mode, with a resolution of 4 cm^−1^ and a scanning number of 16 from 700 to 4000 cm^−1^.

### 2.5. Swelling Test

Pre-weighed, washed and dried samples were immersed in water for 1 h and weighed after removing the excess of water from the surface with filter paper. The swelling degree (%S) was calculated using the following equation:

(2) $$\%S=\frac{\left(W_{s}-W_{d}\right)}{W_{d}}\times 100$$

where W_s_ and W_d_ stand for swollen and dry weight of the samples, respectively.

### 2.6. Thermal Behavior

Differential scanning calorimetry (DSC) analyses were performed using the calorimeter 204 F1 Phoenix (NETZSCH). The samples (~10 mg) were cooled to −100 °C and heated up to 160 °C with a rate of 36 °C min^−1^ under argon atmosphere (50 mL min^−1^) [39]. Thermogravimetric analysis (TGA) was performed using an STA 449 F3 Jupiter balance (NETZSCH). The samples (~10 mg) were heated from room temperature to 500 °C with a heating rate of 20 °C min^−1^ under argon atmosphere (50 mL min^−1^). The glass transition temperature (T_g_) and the degradation temperature (T_d_) were determined following ASTM E1356 and ASTM E473 standards.

### 2.7. Drug Impregnation

The impregnation with IBU was studied with the hydrogel HPG-OA1. Three hydrogel films (~0.5 × 0.5 × 0.2 cm, 0.05 g) were immersed in 2 mL of ethanolic IBU solution with a determined concentration (concentrations of 0.05, 0.5 and 0.75 g mL^−1^ were used) and kept at room temperature without stirring for 24 h. The stability of the hydrogel in ethanol was previously assured. The films were removed from the solutions, rinsed with ethanol and dried at room temperature in a desiccator with silica gel for 24 h and at 60 °C for 30 min in an oven for complete solvent removal. Finally, their surfaces were cleaned gently with filter paper and ethanol to remove IBU crystals that formed on them during the drying step. Morphological analysis of the impregnated samples was performed as described in Section 2.3 to qualitatively evaluate the formation of crystals in the samples.

### 2.8. Drug Release

The release study was done in vitro with the impregnated hydrogel prepared with the 0.5 g mL^−1^ IBU solution. HCl aqueous solution (pH 2) and phosphate buffer (pH 7) were used as release media. The samples (~0.5 × 0.5 × 0.2 cm, 0.05 g) were immersed in 3 mL of the release media in UV-Vis quartz cuvettes at room temperature and placed at a T80 UV-Vis spectrometer (PG Instruments Ltd., Lutterworth, UK). At predetermined intervals, IBU concentrations in the media were analyzed with the spectrometer at 263.8 nm. Calibration curves were performed for both media, and the molar absorptivities were found to be 39.19 m^2^ mol^−1^ at pH 2 and 43.32 m^2^ mol^−1^ at pH 7. The release experiment was performed in triplicate and the cumulative release was calculated using the following equation:

(3) $$Cumulative\;release\%=\frac{m_{t}}{m_{impregnated}}.100$$

where m_t_ is the total mass of the compound released at time t and m_impregnated_ is the total IBU mass impregnated in the sample. Release kinetics and mechanism were evaluated using the Korsmeyer–Peppas model, Equation (4), which can be applied to the data up to 60% of total release [40,41]:

(4) $$\frac{M_{t}}{M}=kt^{n}$$

where M_t_ is the mass released at the time t, M is the mass released at infinite time (which corresponds to m_impregnated_), k is a kinetic constant and n is the release exponent.

## 3. Results

### 3.1. Study of Hydrogel Formation

**Figure 1:**
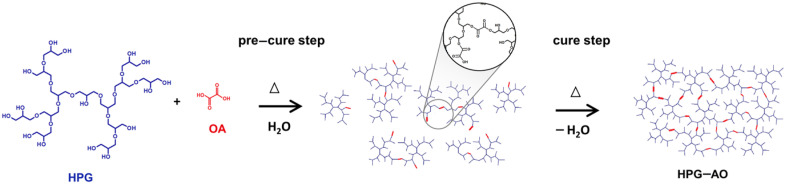
Hydrogel formation schema.

**Figure 2:**
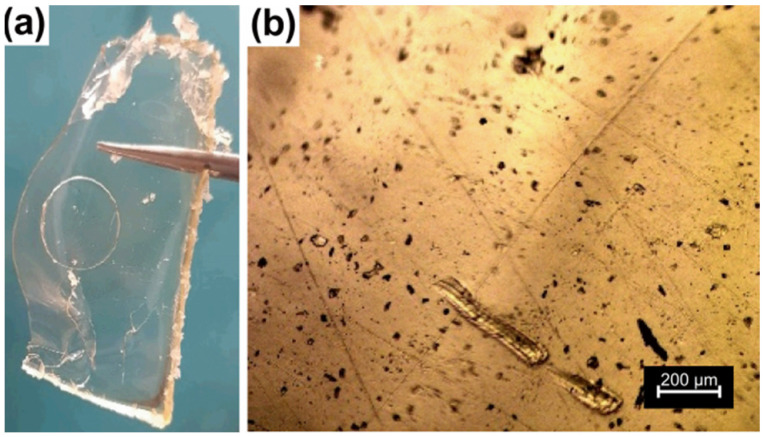
Oxalic acid crosslinked hyperbranched polyglycerol hydrogel HPG-OA1: (**a**) photography and (**b**) optical microscopy image obtained in transmission mode (100×).

The study of the hydrogel formation was conducted under varying conditions to understand the parameters that affect its formation. The proposed formation route is shown in Figure 1. The samples were considered hydrogels when insoluble in water [42], due to their crosslinked structures. Samples 2, 4, 5, 6, 8 and 9 formed hydrogels; while samples 1, 3 and 7 did not. Moreover, sample 3 presented OA crystals in the bulk at the end of the cure time. The hydrogels were obtained as transparent yellowish films that were flexible when dried but fragile when swollen. Figure 2a shows a picture of the HPG-OA1 as an example of the appearance of the dried hydrogels.

Hydrogels could be formed for all tested %OA and media concentrations ([HPG]) depending on the cure temperature and the pre-cure and cure times. When 10% *w*/*w* of OA and [HPG] of 0.13 g mL^−1^ were used, a hydrogel was formed when the cure temperature was 80 °C, sample 2, but not 60 °C, sample 1, even though a longer pre-cure time (18 h) was used. However, increasing the %OA and the [HPG] (20% *w*/*w* of OA and [HPG] of 0.26 and 0.50 g mL^−1^) allowed the hydrogels to be formed using the cure temperature of 60 °C, depending on the pre-cure and cure times. For instance, for the [HPG] of 0.26 g mL^−1^, a hydrogel was formed with 3.0 h of pre-cure time, sample 4, but not with 1.25 h, sample 3, even though sample 3 had a longer cure time (24 h). For the [HPG] of 0.50 g mL^−1^, a hydrogel was formed with 2.3 h of pre-cure time when the cure time was 6.5 h, sample 8, but not when the cure time was 2.5 h, sample 7. The higher [HPG] allowed the formation of the hydrogel within a shorter pre-cure time (samples 8–9 < 4–6) and with higher firmness (sample 8–9 > sample 4–6) accessed qualitatively through appearance and texture; moreover, longer cure times relatively increased the firmness of the hydrogels for the same reaction conditions (samples 4 < 5 < 6 and samples 8 < 9). The higher firmness indicates a higher crosslinking degree [43]. Indeed, Demitri et al. showed that increasing the cure time of their citric acid crosslinked cellulose hydrogels increased their crosslinking degrees [44]. For this reason, samples 6 and 9 were chosen for further characterization. They both represent the two samples with higher crosslinking degrees obtained at 60 °C and formed with two different media concentrations, thus allowing the evaluation of the effect of this concentration on further properties. The crosslinking degree can be accessed indirectly through the %gel of the hydrogels. Samples 6 (HPG-OA1) and 9 (HPG-OA2), presented a %gel of 46.0 and 59.9%, respectively, showing that increasing the media concentration of the reaction increases the crosslinking degree of the hydrogels.

The influence of the reaction parameters on the success of an OA hydrogel formation was also shown by Gohil et al. [45]. They studied the formation of polyvinyl alcohol hydrogels crosslinked with OA and by varying the cure time, cure temperature and %OA, the authors obtained hydrogels with %gel from 22 to 90% [45]. This influence can be explained by the reaction mechanism. For the HPG-OAs, the crosslinking occurs by an esterification reaction between the carboxylic groups of the OA and the hydroxyl groups of the HPG molecules [46]. The esterification is a slow equilibrium reaction that is favored by the remotion of the product or the byproduct, by higher concentrations of the reactants and by temperature [47] Higher temperatures and higher media concentrations increase the speed of the reaction, and the remotion of water shifts its equilibrium to favor the esterification. This explains why the remotion of the water (cure step) was mandatory for the formation of the hydrogels since the water is a byproduct and a solvent of the reaction. However, the remotion of water before the connection of the OA to the HPG molecules, due to a short pre-cure time, leads to OA crystallization during the cure step, which makes this molecule no longer act as a crosslinking agent to complete the gel formation; this was seen for sample 3, which did not form a hydrogel. On the other hand, if the pre-cure time is enough to form HPG-OA structures, during the cure step these structures can continue to connect over time, leading to gel formation and increasing the crosslinking degree.

### 3.2. Optical Microscopy

The morphology of the sample HPG-OA1 was studied using optical microscopy in transmission mode, Figure 2b. It was transparent to light and presented some dispersed pores, which evidenced that the material had low porosity and was amorphous [48]. The few observed pores are related to small bubbles formed during the mixing of the reaction media, pre-cure and/or cure steps. The compact structure of the hydrogel was a result of a mesh size decrease resulting from the increase of the crosslinking density during the cure step and the strong pore contraction as the water slowly evaporated in the oven during the cure step. Indeed, evaporation, as a drying technique, is intended to generate compact structures. Buchtova et al., for instance, studied the morphology of hydrogels dried using different techniques and showed that when evaporation was used a dense material with almost no porosity was obtained, in contrast to freeze drying or supercritical drying when opaque porous materials with low density were obtained [49].

### 3.3. Fourier Transformed Infrared Spectroscopy

**Figure 3:**
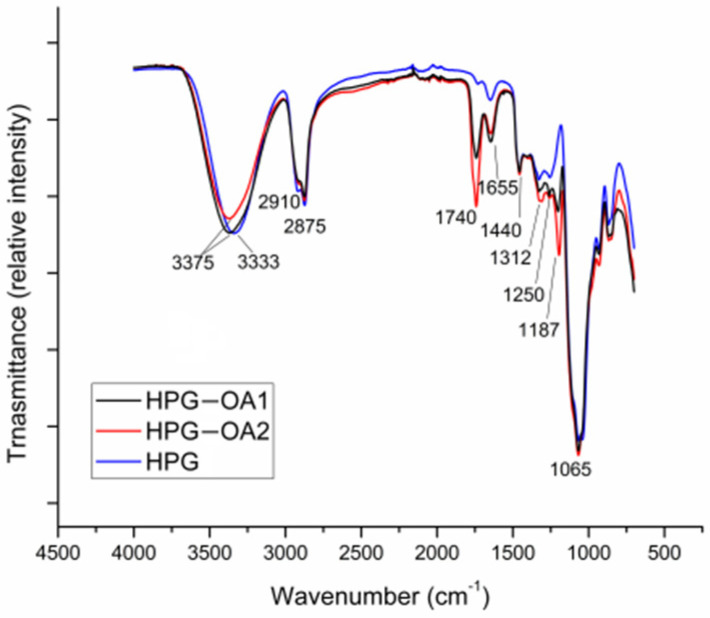
FTIR spectra of HPG-OA1, HPG-OA2 and hyperbranched polyglycerol (HPG). The main peaks associated with the structures are highlighted.

FTIR spectra of HPG-OA1, HPG-OA2 and HPG are shown in Figure 3. Hydrogels and HPG spectra show a band of stretching of their hydroxyl groups from 3000 to 3687 cm^−1^ [46,50]; this band exhibits a clear loss of intensity for the HPG-OA2, indicating a reduction in its number of hydroxyl groups when compared to HPG, due to their esterification. The stretching band of C-H bonds was observed from 2800 to 3000 cm^−1^ for all samples [51]. HPG-OA1 and HPG-OA2 spectra presented a band at 1740 cm^−1^ and a band at 1187 cm^−1^ related, respectively, to C=O and C-O stretching of the aliphatic esters produced by the esterification reaction; their intensities are higher for HPG-OA2 in comparison to HPG-OA1. The bands at 1655 cm^−1^ and 1250 cm^−1^ are related, respectively, to the C=O and C-O stretching of unreacted pendant carboxylic groups; their intensities are higher for HPG-OA1 in comparison to the HPG-OA2. This pendant group is present in the hydrogel structures when some OA molecules connect to it through the reaction of only one of their two carboxylic groups. The absorption at 1000–1150 cm^−1^ is related to C-O stretching of the ether groups present in the polyglycerol backbone [50,51] and all samples present this band with similar intensities.

The results, therefore, are evidence of the crosslinking of the HPG with OA through ester bonds to form the hydrogels and that HPG-OA2 presents a higher crosslinking degree when compared to HPG-OA1, which is in accordance with their %gel results and characteristic firmness.

### 3.4. Swelling Test

**Figure 4:**
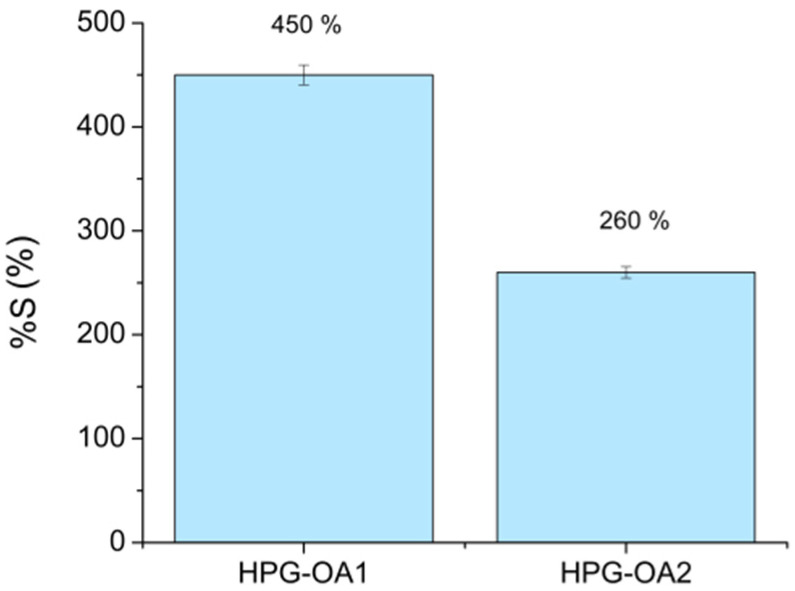
%S of HPG-OA1 and HPG-OA2.

The swelling degrees (%S) of HPG-OA1 and HPG-OA2 are presented in Figure 4. These %S are within the wide range presented by the literature [52]. When swollen, the hydrogels exhibited some fragility, with HPG-OA2 being more fragile than HPG-OA1. The ability of the hydrogels to absorb water is related to their hydrophilicity, free volume and chain flexibility. The studied hydrogels are hydrophilic due to the presence of the hydroxyl groups from the HPG and the pendant carboxylic groups from the OA [51], which readily establish hydrogen bonds with the water molecules. Moreover, hydrogels are amorphous and the hyperbranched structure of the HPG formed by single bonds gives chain flexibility and high free volume to the hydrogel. Conversely, the flexibility and free volume decrease as the degree of crosslinking increases, reducing the mesh size of the structure, which is the reason why HPG-OA2 presents a lower swelling degree and higher fragility than HPG-OA1 [53]. Indeed, the %S varies with the structure of the hydrogel. Gohil et al., for instance, obtained %S from 270 to 1700% for their hydrogels based on polyvinyl alcohol and OA with a range from high to low crosslinking degrees, indicated by %gel from 90 to 22% [45].

### 3.5. Thermal Behavior

**Figure 5:**
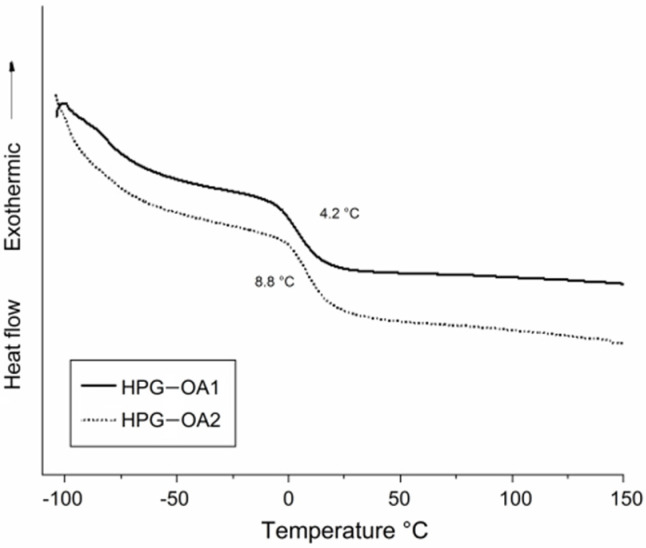
DSC thermograms of HPG-OA1 and HPG-OA2.

The thermal behavior of the HPG-OA1 and HPG-OA2 was studied through DSC and TGA. Their DSC thermograms are presented in Figure 5, which shows that the T_g_ temperatures for HPG-OA1 and HPG-OA2 are, respectively, 4.2 and 8.8 °C and that there were no fusion peaks, confirming that the hydrogels were amorphous. The pure HPG shows a couple of T_g_ events in the temperature range of −50 to −20 °C, referent to the mobility of different regions of the hyperbranched structure [50]. The presence of single T_g_ events for the hydrogels, and with higher values, points to the observed reduction in their chain mobility caused by the crosslinking. Moreover, HPG-OA2 presents a higher T_g_, confirming its higher crosslinking degree.

**Figure 6:**
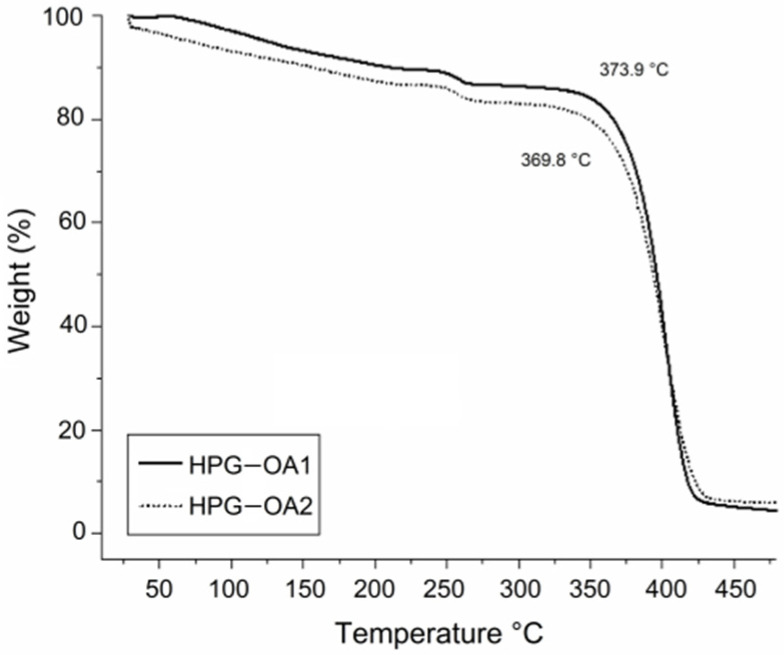
Thermogravimetric curves of HPG-OA1 and HPG-OA2.

The thermogravimetric curves of hydrogels are presented in Figure 6. The samples presented three weight loss events. The first was in the temperature range of 50 to 200 °C, 10.2 and 13.3%, for HPG-OA1 and HPG-OA2, respectively, due to the loss of water molecules trapped in the structure by hydrogen bonds [45]. The second event was verified in the temperature range of 230 to 280 °C, 2.9 and 3.4%, respectively, which can be attributed to the loss of the OA pendant groups [54,55]. The third and main weight loss event was observed in the temperature range of 300 to 490 °C, 82.7 and 77.4%, respectively, due to the decomposition of ester linkages and the crosslinked polymer backbone [54]. This third thermal event was the one considered to determine the T_d_ of the hydrogels, 373.9 and 369.8 °C, respectively, since it is the event that reflects the decomposition of the hydrogel structure and the effect of crosslinking on the final material. These T_d_s are higher than the T_d_ for this type of HPG, around 300 °C [50], as a result of the crosslinking, which leads to an increase in the thermal stability of polymers [56,57].

### 3.6. Drug Impregnation

The HPG-OA1 hydrogel was selected for the drug impregnation study with the model drug, IBU, due to its lower fragility when swollen, compared to HPG-OA2. The impregnation was done in ethanol due to the high solubility of IBU in this solvent (0.88 g mL^−1^, 25 °C) [58,59].

**Figure 7:**
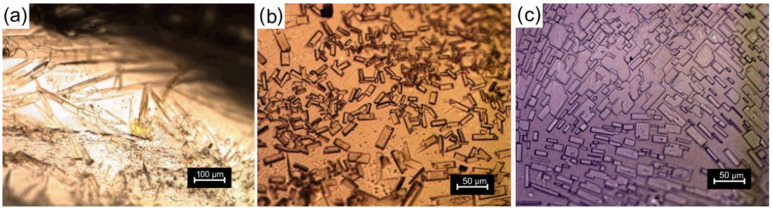
Optical microscopy images in transmission mode of HPG-OA1 hydrogels impregnated in ibuprofen (IBU) solutions of (**a**) 0.05 g mL^−1^ (200×), (**b**) 0.5 g mL^−1^ (400×) and (**c**) 0.75 g mL^−1^ (400×).

**Figure 8:**
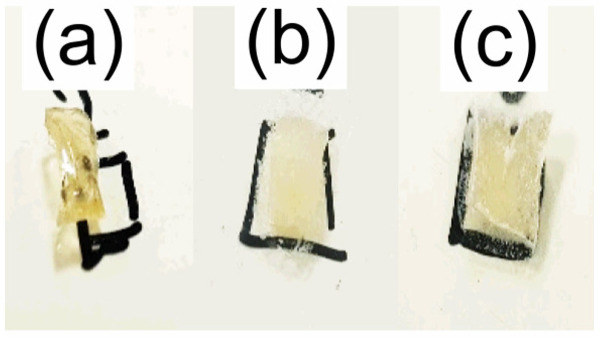
Photographs of the impregnated HPG-OA1 samples after the drying step. Samples impregnated in IBU solutions of (**a**) 0.05, (**b**) 0.5 and (**c**) 0.75 g mL^−1^.

The morphology of the impregnated samples was studied via optical microscopy and is shown in Figure 7. It was observed that all impregnated samples presented crystals dispersed in their matrix and can be classified as matrix-encapsulated systems [60]. The amount of crystals increased and the shape of the crystals varied with increasing concentration of the ethanolic IBU solution. Moreover, the samples impregnated in ibuprofen solutions of 0.5 and 0.75 g mL^−1^ presented crystals on their surface after the drying step as shown in Figure 8.

The dispersion of the drug in the form of crystals is a result of the moderate interaction of the IBU with the structure of the hydrogel. IBU presents two main sites of interaction, the aromatic ring and the carboxylic group [61]. In a previous work, it was shown through a nuclear magnetic resonance study that IBU preferentially interacts with the carbonyl groups of acetylated HPG over the HPG core, due to their polarity [50]. It can be understood, therefore, that the IBU intermolecular interactions, which allow molecular drug-chain association are restricted to the crosslinking regions and to the OA pendant groups of the hydrogel structure, and that the excess of IBU molecules establish drug–drug interaction forming crystals, as the solvent is removed. The crystals presented parallel faces; the ones formed from the lower concentration solution (0.05 g mL^−1^) presented a needle shape, while the others presented a parallelepiped or plate shape. These different shapes have been reported in the literature and are associated with the specific interactions that occur between the IBU molecule and the crystallization environment [62,63], which changes with the change in the IBU concentration. Indeed, it is seen that higher IBU concentrations lead to a larger number of nucleation sites in the hydrogel and a limitation of the crystal size as they approach each other. The presence of IBU crystals on the surface of the samples may be the result of the molecular transport of IBU by ethanol to the surface of the sample during the drying step.

### 3.7. Drug Release

**Figure 9:**
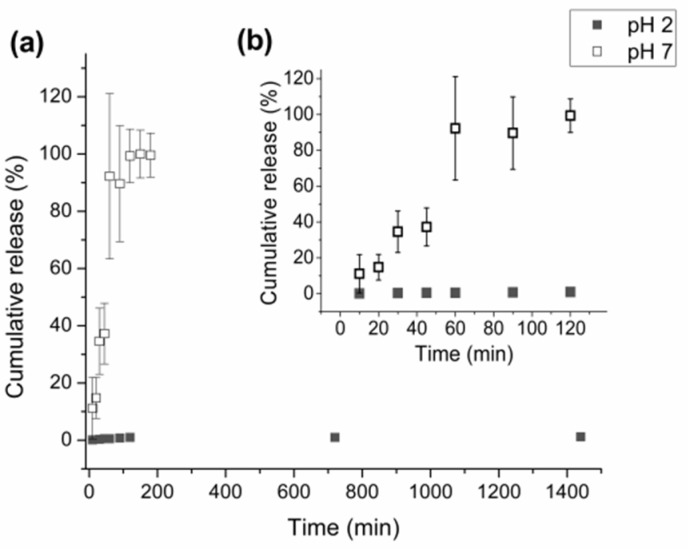
Release profiles of the IBU from the hydrogel impregnated with a 0.5 g mL^−1^ IBU solution; (■) at pH 2 and (□) at pH 7: (**a**) 0–1440 min and (**b**) 0–120 min.

**Figure 10:**
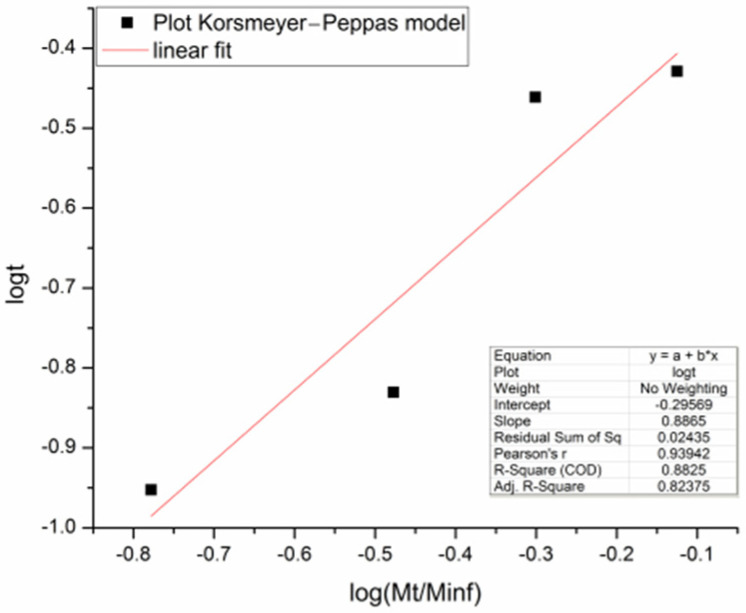
Korsmeyer–Peppas plot for the release at pH 7.

**Figure 11:**
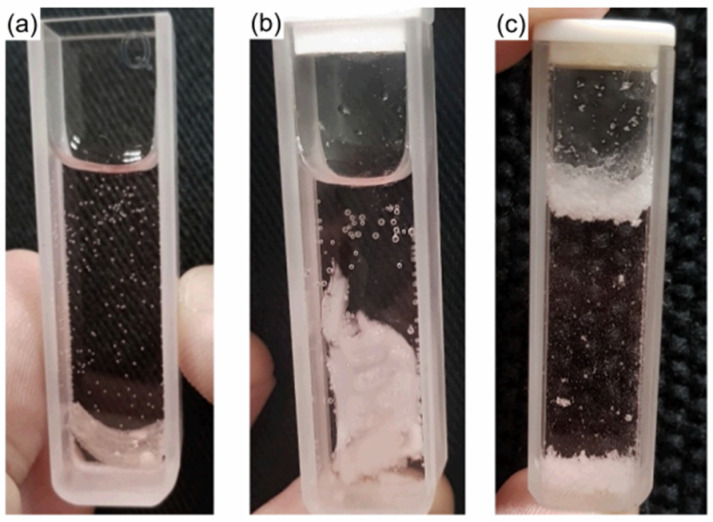
Degradation of the impregnated HPG-OA1 in pH 7 (**a**) t = 0 min, (**b**) t = 120 min, (**c**) t = 180 min.

Release profiles at pH 2 and pH 7 for the HPG-OA1 hydrogel impregnated in a 0.5 g mL^−1^ IBU solution are presented in Figure 9. The release at pH 2 was negligible when compared with the release at pH 7 and because of that, the experiment was carried out for 1440 min; however, the concentration of the solution stabilized after 120 min (0.003 ± 0.001 mg mL^−1^). The release at pH 7 was carried out for 180 min and its profile was treated using the Korsmeyer–Peppas model, Figure 10, and showed an *n* = 0.89, which indicates a release mechanism of anomalous transport that occurs when the release is promoted by the erosion and/or degradation of the matrix [40]. It was observed that at this pH, the samples degraded and eroded during the experiment, resulting in the formation of small particles at 180 min, Figure 11. The concentration of the media stabilized after 60 min (0.24 ± 0.02 mg mL^−1^), showing that initially the degradation led to an increase in the mesh size of the crosslinked structure, which allowed the release of IBU before the erosion takes place, leading to the formation of the small particles. Because of that, the release was considered complete and the impregnated amount of IBU in the hydrogel could be determined as 15 µg g^−1^. The release at pH 2 was not modeled due to the low release of IBU in this medium; moreover, no degradation was observed at this pH. It is important to note, especially for pH 2, that the release was not limited by the media concentration as they did not get close to saturation (solubility of IBU at pH 1.4: 0.036 mg mL^−1^, 20 °C, at pH 2: 0.053 mg mL^−1^, 37 °C, at pH 7.4: 6.14 mg mL^−1^, 20 °C and at pH 7: 3.89 mg mL^−1^, 37 °C [64,65]), which emphasizes the role of the degradation in the release.

It was possible to verify that the studied hydrogel presents a pH-sensitive degradation [5], which was responsible for the release of the IBU at pH 7. The hydrogel responded to pH by degrading at pH 7, but not at pH 2, through the hydrolysis of their ester bonds. Paris et al., for instance, synthesized hydrogels with a crosslinker bearing an ester group that was also hydrolysable at high, but not at low pHs [66]. The pH-sensitive degradation for ester crosslinked hydrogels can be explained by the speed of ester hydrolysis in different media; Mabey et al. showed in their review that the rate constant of ester hydrolysis varies with pH and that for highly activated esters, the hydrolysis at pH 7 is more rapid than the acid-catalyzed process at low pH [30]. Oxalic acid generates highly activated ester bonds, due to their side-by-side carboxylic groups [67], which explains the observed degradation behavior of hydrogel.

The pH-sensitive degradation of the HPG-OA can be explored for oral drug administration applications targeting release in the intestines [68,69], since the pH of the stomach is ca. 2 and the pH of the intestine varies from 6.5 to 7.4 [70].

## 4. Conclusions

A novel OA crosslinked HPG hydrogel was obtained which presented a pH-sensitive degradation behavior that triggered the release of IBU at pH 7. The parameters involved in the formation of the HPG-OA hydrogel were understood, showing that a pre-cure step was crucial and that an increase in the reactants’ concentrations increased the crosslinking degree of the hydrogel expressed through a higher %gel and lower %S. The hydrogel could be impregnated, forming a matrix-encapsulated system with IBU. The use of OA as a crosslinker generated ester bonds that are highly activated for hydrolysis at neutral pH, leading to the matrix degradation at pH 7, but not at pH 2. This behavior promoted a pH-sensitive drug release that can be applied for oral administration of drugs targeting the release in the intestines. HPG-OA is, therefore, an intelligent hydrogel that can be easily formed and can largely benefit pharmaceutical and biomedical fields for controlled drug release applications.

## Figures and Tables

**Table 1:** Parameters of the study of hydrogel formation.

Sample	OA (% *w*/*w*)	[HPG] (g mL^−1^)	Pre-Cure	Cure	
			t (h)	t (h)	T (°C)
1	10	0.13	18	24	60
2	10	0.13	2.5	24	80
3	20	0.26	1.3	24	60
4	20	0.26	3.0	3.7	60
5	20	0.26	3.0	7.7	60
6 (HPG-OA1)	20	0.26	3.0	13.5	60
7	20	0.50	2.3	2.5	60
8	20	0.50	2.3	6.5	60
9 (HPG-OA2)	20	0.50	2.3	13.5	60

## Data Availability

The data presented in this study are available in “pH-Sensitive Degradable Oxalic Acid Crosslinked Hyperbranched Polyglycerol Hydrogel for Drug Controlled Release” and its supplementary material.
